# Permanent and Persistent Atrial Fibrillations Are Independent Risk Factors of Mortality after Severe COVID-19

**DOI:** 10.3390/jcm13113112

**Published:** 2024-05-26

**Authors:** Agnieszka Zając, Ewa Wrona, Jarosław D. Kasprzak

**Affiliations:** 1 I Department of Cardiology, Medical University of Lodz, Bieganski Hospital, Kniaziewicza 1/5, 91-347 Lodz, Poland; 2 Department of Clinical and Laboratory Genetics, Medical University of Lodz, 92-213 Lodz, Poland; 3 Department of Chemotherapy, Medical University of Lodz, Copernicus Memorial Hospital, 92-213 Lodz, Poland

**Keywords:** atrial fibrillation, COVID-19, mortality risk factors

## Abstract

**Background:** The new coronavirus disease (COVID-19), a pandemic infection caused by severe acute respiratory syndrome coronavirus (SARS-CoV-2), had a deep global influence on morbidity and mortality profiles. Comorbidities, especially cardiovascular diseases, were identified to strongly modify the clinical course of COVID-19. However, the prognostic role of incident or prevalent atrial fibrillation has not been fully explained. The aim of this study was to evaluate the association between atrial fibrillation and outcomes following hospitalization in patients with severe COVID-19. **Methods:** We analyzed 199 patients (72 female, median age 70 years) with severe COVID-19 hospitalized between November 2020 and February 2021, due to SARS-CoV-2 infection. The study cohort included 68 patients with a history of AF (34 patients with paroxysmal AF, 19 with permanent AF, 15 patients with persistent AF), and 51 patients presented with AF during hospitalization. **Results:** Overall mortality during 90 days from the admission to hospital was 41% (*n* = 82). Non-survivors were older, had significantly elevated inflammation markers (CRP, WBC, procalcitonin, IL-6), NT-proBNP and D-dimer on the first day of hospitalization, lower left ventricular ejection fraction and worse kidney function, as compared to those who stayed alive during the follow-up. Among the hospitalized patients with COVID-19, a history of AF and the presence of AF during hospitalization contributed to higher mortality. Patients with permanent and persistent AF were at the highest risk of death. Different presentations of AF (any history of AF, the subtypes of AF—paroxysmal, permanent, persistent—and the presence of AF during hospitalization) were included in multivariate analysis, aiming to identify independent risk factors of death in the study period. We found that AF was related to worse prognosis, and persistent or permanent forms represented an independent predictor of mortality. **Conclusions:** Different clinical presentations of AF have varying impacts on survival in severe COVID-19. Mortality in hospitalized patients with severe COVID-19 was higher among patients with a history of AF, especially with persistent and permanent types of AF, and with AF present during hospitalization.

## 1. Introduction

Coronavirus disease (COVID-19) was declared a global pandemic by the World Health Organization in March 2020. COVID-19, caused by novel severe acute respiratory syndrome coronavirus (SARS-CoV-2), emerged as a global health threat and deeply influenced morbidity and mortality throughout the world [1]. Although most patients with COVID-19 develop mild symptoms, some develop a severe form of the disease. The impact of concomitant cardiovascular risk factors and cardiovascular disease (CVD) on the clinical course of COVID-19 has been studied worldwide [2,3,4,5,6]. CVD risk factors, such as obesity, hypertension and diabetes, were identified to worsen the clinical course of COVID-19. Furthermore, not only CVD risk factors but most types of cardiovascular disease worsen the prognosis of COVID-19. In a recent meta-analysis of 307,596 patients with COVID-19, 15.1% with CVD showed that CVD was associated with a 4-fold higher risk of mortality (OR, 4.33; 95%CI, 3.16–5.94) [3]. Docherty et al., using one of the largest cohort studies of hospitalized patients in the United Kingdom (*n* = 20.133), indicated that chronic cardiac disease was significantly connected with mortality (adjusted hazard ratio (HR) 1.16) [4]. Another study in the USA also found a relationship between CVD and COVID-19-related death [5]. Moreover, it is known that SARS-CoV-2 virus can affect the cardiovascular system, cause thromboembolic disease, myocarditis, heart failure or vasculitis, similar to Kawasaki syndrome, and the unfavorable cardiac consequences worsen the prognosis [6,7,8]. The impact of atrial fibrillation (AF), one of the most common types of CVD, on COVID-19 mortality remains uncertain. The aim of our study was to evaluate the strength of the influence of AF on mortality in patients hospitalized due to severe COVID-19 and to analyze the prognostic impact of different clinical forms of AF.

## 2. Materials and Methods

We retrospectively analyzed a cohort of consecutive patients hospitalized in I Department of Cardiology Medical University of Lodz (temporarily converted into an infectious disease department, dedicated to severe COVID-19 treatment) between October 2020 and February 2021. COVID-19 diagnosis was established based on the RT-PCR result, and the treatment followed current WHO recommendations. Comorbidities of potential prognostic importance were determined based on the prehospital medical history and diagnostic tests performed during ongoing hospitalization. Glomerular filtration rate was estimated according to MDRD equation. The designated endpoint was death within 90 days from the COVID-19-related admission to hospital.

### 2.1. Study Population

We analyzed 199 patients (72 female, median age 70 years) with COVID-19 hospitalized in Cardiology Department between November 2020 and February 2021, due to SARS-CoV-2 infection. Study population presented with severe COVID-19 with pneumonia and desaturation <94% (except for patients requiring initial ECMO), with severe cardiovascular comorbidities. The severe course of COVID-19, defined as saturation below 90%, lung involvement above 50% in computer tomography, the need for high-flow oxygen therapy or intubation, was observed in 98 patients (49%). Thus, 68 (34%) patients had preceding history of AF, and 51 (26%) presented AF in ECG recorded during the index hospitalization (incident AF). This study is a retrospective analysis of “all-comer” hospital records, and no additional exclusion criteria existed.

### 2.2. Statistical Analysis

Continuous data were presented as medians with interquartile ranges (IQRs). For comparisons of two or multiple groups, Mann–Whitney *U*-test, Pearson’s chi-squared test and Kruskal–Wallis test were used where applicable. Dunn’s multiple comparison test was applied for nonparametric post hoc analysis. Overall survival times (time from hospital admission to death of any reason) were presented with Kaplan–Meier curves and analyzed using log-rank test. Cox’s proportional hazard regression was used for overall survival analysis. A multivariate regression analysis was conducted for factors identified in the univariate analysis. Statistica 13.1 (Dell Inc., Round Rock, TX, USA) was used for all computations. A *p*-value lower than 0.05 was considered statistically significant. For graphical results’ presentation, GraphPad Prism 9 software (GraphPad Software, La Jolla, CA, USA) was used.

## 3. Results

### 3.1. Subsets of Patients with AF

Among 68 patients (pts) with a history of AF (median age 75 years, 28 female), the most prevalent form was paroxysmal AF (34 pts), followed by permanent AF (19 pts) and persistent AF (15 pts). In addition, we identified a subset of 51 pts with AF present during the COVID-19 hospitalization. As compared to patients without a history of AF (AF negative), those with AF (AF positive) were older (*p* < 0.001), characterized by a lower left ventricular ejection fraction (LVEF 49% vs. 55%, *p* = 0.001) and higher NT-proBNP level on admission (*p* < 0.001), Table 1. Moreover, a trend to a higher incidence of chronic kidney disease (CKD, stage 3 or higher) was observed in AF-positive patients. The markers of the high-risk COVID-19 course—need for high-flow oxygen therapy or intubation, oxygen saturation <90% and lung involvement >50% in computer tomography—were similarly distributed in subsets with 36/68 and without AF 62/131, *p* = 0.45. The prevalence of comorbidities was comparable in the subgroups.

Hospital therapies during COVID-19 were similar between the subgroups. COVID-19-specific therapy was introduced according to the availability and standard criteria with 21/131 AF-negative patients and 6/68 AF-positive patients, without significant differences between the groups.

Anticoagulation was initiated in all patients with AF on admission to the hospital. According to the clinical condition and previous medication, different anticoagulants were used (Table 1, 33 patients used oral anticoagulants before the hospitalization). In addition, each patient hospitalized due to COVID-19 was treated with prophylactic or intermediate intensity anticoagulation, depending on the clinical picture. Treatment for pulmonary embolism was the major cause for the implementation of therapeutic anticoagulation in patients without a history of AF. The uniform anticoagulation protocol was used at hospital discharge in pts with an AF diagnosis.

The characteristics of the subsets defined according to the clinical form of AF are presented in Table 2 and Table 3.

### 3.2. Predictors of Death According to AF Status

**Figure 1:**
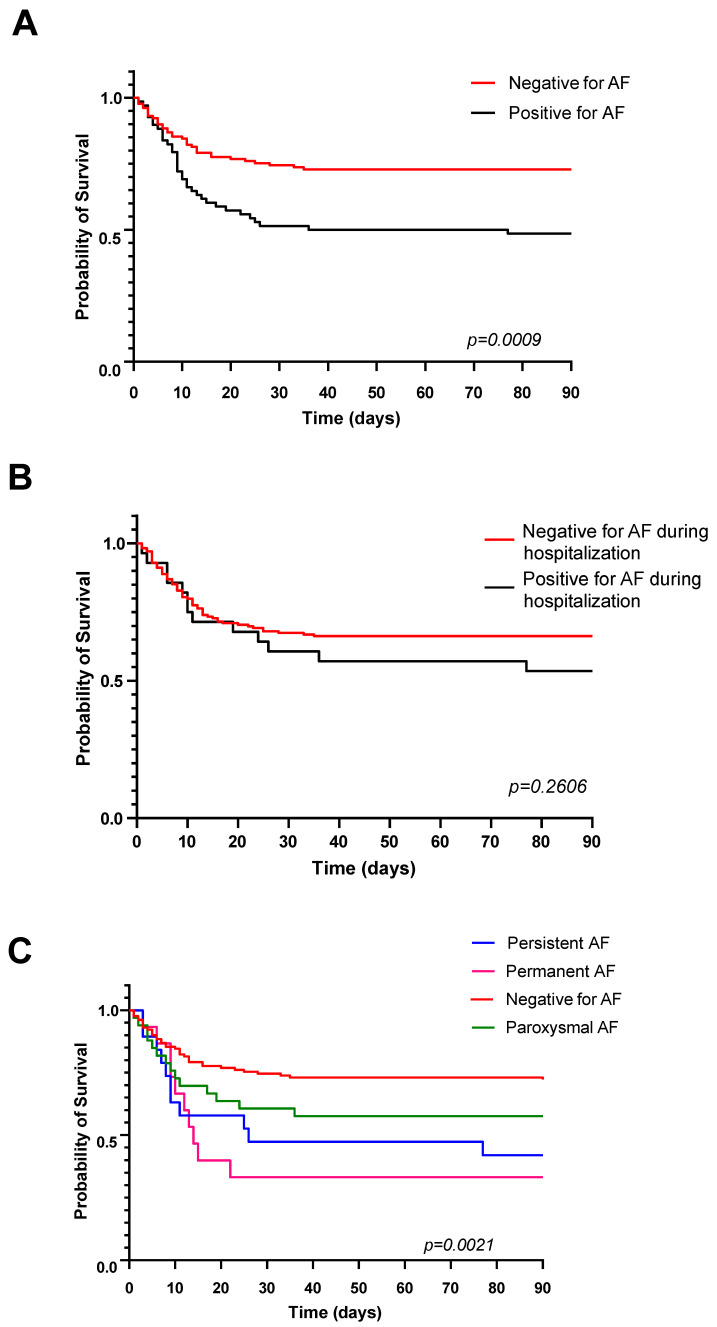
Kaplan–Meier curves demonstrating worse 90-day survival in patients with any evidence of atrial fibrillation (**A**), with no significant influence of atrial fibrillation recorded during the hospital stay only (**B**). Bottom plot (**C**) illustrates differential survival in patients with permanent, persistent and paroxysmal atrial fibrillation.

Overall, mortality during 90 days from the admission to hospital was 41% (82 pts) and 56% (38/68 pts) among those with a history of AF. AF represented a significant univariate predictor of death (*p* = 0.0025). The clinical types of AF had differential influences on mortality, ranging from 44% (15/34 pts) among pts with paroxysmal AF to 67% (10/15) in pts with persistent AF and 68% (13/19) in pts with permanent AF (*p* = 0.004). The presence of AF during hospitalization was also related to a higher 90-day mortality: 61% (31/61 pts) versus 34% (51/148) among pts free of AF during the hospital stay, *p* = 0.001. Survival analysis using Kaplan–Meier curves confirmed that any past or current evidence of AF increased the 90-day mortality (Figure 1) and that the risk varied between different forms of AF, with the highest risk in the permanent AF subgroup. The detection of AF during the hospital stay did not significantly modify 90-day mortality according to Kaplan–Meier curves.

### 3.3. Identification of Prognostic Factors of Death

Based on all demographic, clinical and echocardiographic parameters, univariate analysis was performed to identify the predictors of death. Table 4 presents a comparison of the individual parameters in the group of patients who died within 90 days vs. the survivors; only statistically significant values are presented.

People who died were older and had significantly higher inflammation markers (CRP, WBC, procalcitonin, Il-6), NTpro-BNP and D-dimer levels on the first day of hospitalization, lower left ventricular ejection fraction and worse kidney function compared to survivors. Among the non-survivors, a history of AF was significantly more prevalent.

### 3.4. Independent Risk Factors of Death

Based on univariate predictors, three models of multivariable logistic regression were created to identify independent predictors of death using different definitions of AF burden. Specifically, we incorporated dichotomous information regarding whether a patient had a history of AF (whether diagnosed with permanent or persistent AF) and whether the AF was present during hospitalization. The best fit was obtained in the second model (with various subtypes of AF), and, thus, the independent AF contribution for the prediction of mortality was inferred by persistent or permanent but not paroxysmal forms. Moreover, older age, a worse glomerular filtration rate, higher NT-proBNP and the white blood cell level were incorporated in the models as independent predictors of overall mortality after COVID-19 (Table 5, Table 6 and Table 7).

## 4. Discussion

The main finding of our study on patients with severe COVID-19 is that persistent and permanent AF is an independent risk factor for infection-related mortality. The presence of AF, either in history or during hospitalization, is a marker of worse prognosis but not independent predictors of death.

Atrial fibrillation is a well-documented CV risk factor in the general population; however, the impact of AF on the clinical course of COVID-19 has not been adequately defined. Since the earliest published reports, the presence of pre-existing cardiovascular disease has been associated with a high risk of adverse COVID-19 outcomes, and cardiovascular complications during the infection are common [3,9,10]. Our results highlight the prognostic relevance of AF—34% of COVID-19 hospitalized patients had a history of AF associated with a fatal outcome. Permanent and persistent subtypes of AF carried the greatest risk of death, and the presence of AF during hospitalization worsened the survival. Consistent with previous studies, non-survivors were older, had significantly higher inflammation markers (CRP, WBC, procalcitonin, Il-6), NTpro-BNP and D-dimer levels at admission, lower left ventricular ejection fraction and worse kidney function compared to survivors [11]. Moreover, older age, worse glomerular filtration, higher NT-proBNP and white blood cells were independent predictors of death from any cause. Identifying the risk factors of the severe clinical course of COVID-19 may be important for optimized treatment, with a potential influence on the risk of mortality [11,12]. Existing data on the prognostic impact of AF in COVID-19 are not consistent, indicating correlation with adverse events. In some studies, AF, especially new-onset AF, was proposed as an independent predictor of in-hospital all-cause mortality and cardiovascular death [13,14]. Another analysis also revealed a significant correlation of new-onset AF with a higher risk of all- cause mortality among COVID-19 patients [15], whereas Rosenblatt et al. suggested that new-onset AF in COVID-19 hospitalized patients may be a marker of an adverse clinical profile rather than an independent predictor of mortality [16]. Another study highlighted the existing heterogeneity in the strength of association between heart disease subtypes and in-hospital mortality, with the strongest association for heart failure (95% CI 1.10–1.30; *p* < 0.018) [16] In line with other studies, we found that AF was a marker of poor prognosis [9,11,12,13,14,15,16,17,18]. Both the history and incident AF in COVID-19 were related to mortality, representing a simple clinical variable suitable for early risk stratification.

Importantly, COVID-19 may provoke paroxysmal AF [17]. A link between COVID-19 and new-onset AF and AF worsening is visible, and we can find it in the scientific literature. Li Z et al. showed in their meta-analysis that 11% of hospitalized COVID-19 patients presented with new-onset AF. Elderly age along with European and American ethnicity were found as risk factors for new-onset AF [15]. Pathophysiological patterns involved in the development of new-onset AF after COVID-19 can lead to atrial remodeling, which can further perpetuate arrhythmia, although in the majority of patients, AF remains paroxysmal [18,19]. Moreover, it has been speculated that atrial arrhythmias may represent a signal of myocardial involvement in COVID-19 disease, which may lead to worse survival [20,21,22,23]. SARS-CoV-2 virus may induce cardiac complications, myocardial damage or myocarditis, as evidenced by elevated troponin levels and abnormal cardiac magnetic resonance findings. Kapusta et al. reported that QRS fragmentation (*p* = 0.031), arrhythmias (atrial fibrillation, supraventricular extrasystole, ventricular extrasystole) (*p* = 0.008) and male gender (*p* = 0.007) were independently associated with myocardial dysfunction after COVID-19, which may represent a valuable marker for physicians [24]. Our study adds to the understanding of AF in COVID-19 in well-defined groups of patients with severe pneumonia and high CVD burden, and we propose the different prognostic significance of persistent/permanent types of arrythmia. Our results suggest that simple clinical findings of persistent/permanent AF may help to identify patients with an elevated risk of short-term mortality. This is especially important in the setting of excess cardiovascular mortality during the pandemic era and disruption of healthcare services [25,26,27].

Cardiovascular complications in COVID-19 seem to be a crucial prognostic factor, so finding novel predictors and therapeutic targets of myocardial damage is vital, including molecular biology advances. Izzo C et al., in their study, proposed miRNAs in the human genome as valuable predictors of cardiac and vascular damage in COVID-19. Moreover, miRNAs may predict COVID-19 progression, some symptoms, treatment response and long- term cardiovascular consequences [28]. The SARS-CoV-2 pandemic had a deep global influence on morbidity and mortality profiles, so further research for precise biomarkers, prevention and treatment should be conducted worldwide.

### Limitations

Our study has methodological limitations. This is a single-center, all-comers study, initiated in the early stage of the pandemic, when existing evidence did not allow for formal sample size calculations. The sample size is, therefore, limited.

## 5. Conclusions

In our study of a cohort with severe COVID-19, mortality was higher among patients with a history of AF, incident AF and especially with persistent and permanent types of AF, which represented an independent risk factor for 90-day mortality.

## Figures and Tables

**Table 1:** Characteristics of patients with (AF positive) and without history of atrial fibrillation (AF negative).

Characteristics	All *N* = 199	AF Negative *N* = 131	AF Positive *N* = 68	*p* Value
Age (years)	70 (61–77)	67 (55–74)	75 (69–81)	<0.001
Gender (female)	72 (36%)	44 (34%)	28 (41%)	0.29
Body mass index (kg/m^2^)	28 (25–32)	27.9 (24.9–32.2)	27.6 (25–31)	0.66
C-reactive protein—day 1 (mg/L)	54 (12–119)	52 (10–114)	70.6 (19–124)	0.23
Interleukin-6—day 1 (pg/mL)	42 (12–82)	38 (10–75)	47.5 (24–104)	0.12
White blood cells—day 1 (n/µL)	7.4 (5.5–10.5)	7.20 (5.4–11)	8.1 (6.2–10.6)	0.4
Procalcitonin—day 1 (ng/mL)	0.1 (0.07–0.3)	0.11 (0.06–0.2)	0.18 (0.09–0.6)	0.003
NT-proBNP—day-1 (pg/mL)	1334 (347–4399)	846 (150–2292)	3340 (1073–10214)	<0.001
D-dimer—day 1 (µg/L)	1534 (725–4413)	1224 (691–2780)	2413 (749–4753)	0.08
Left ventricular ejection fraction (%)	54 (42–60)	56 (47–60)	49 (35–58)	<0.001
Chronic kidney disease (CKD 3–5)	88 (44%)	43 (33%)	45 (66%)	<0.001
Glomerular filtration rate—day 1	63 (42–88)	74 (53–97)	48 (36–69)	<0.001
Severe course of COVID-19	98 (49%)	62 (47%)	36 (53%)	0.45
Initial saturation—day 1 (%)	93 (90–97)	93 (90–97)	90 (88–95)	0.17
Need for high-flow oxygen	15 (8%)	12 (9%)	3 (4%)	0.21
Need for intubation	38 (19%)	23 (18%)	15 (22%)	0.45
Pneumonia in computer tomography >50% lung volume	18 (9%)	10 (8%)	8 (12%)	0.34
Hypertension	142 (71%)	93 (74%)	49 (75%)	0.6
Chronic coronary syndrome	89 (45%)	56 (42%)	33 (48%)	0.53
Diabetes	58 (29%)	37 (28%)	21 (31%)	0.54
Nicotinism	27 (13%)	19 (15%)	8 (12%)	0.55
90 days—mortality	82 (41%)	44 (34%)	38 (56%)	0.0025
Medications during hospitalization:				
LMWH	165 (83%)	129 (98%)	36 (52%)	<0.001
UFH	12 (6%)	2 (1.5%)	10 (15%)	<0.001
Oral anticoagulants	54 (27%)	32 (24%)	22 (32%)	0.4
Covid-specific therapy	27 (14%)	21 (16%)	6 (9%)	0.19

AF—atrial fibrillation, LMWH—low-molecular-weight heparin, NT-proBNP—N-terminal prohormone of brain natriuretic peptide, UFH—unfractionated heparin.

**Table 2:** Characteristics of subgroups with different clinical forms of atrial fibrillation (AF paroxysmal vs. permanent vs. persistent).

Characteristics	Paroxysmal AF *N* = 34	Persistent AF *N* = 15	Permanent AF *N* = 19	*p* Value
Age (years)	74 (70–77)	71 (65–80)	80 (69–84)	<0.001
Gender (female)	15 (44%)	6 (40%)	7(37%)	0.71
Body mass index (kg/m^2^)	27 (24–29)	29 (26–34)	28 (26–35)	0.18
C-reactive protein—day 1 (mg/L)	91 (34–133)	17 (6–71)	68 (21–124)	0.06
Interleukin-6—day 1 (pg/mL)	51 (37–113)	36 (6–113)	47 (2–89)	0.16
White blood cells—day 1 (n/µL)	8 (4.5–10.5)	8.8 (7–117)	7.2 (6.1–10.6)	0.47
Procalcitonin—day 1 (ng/mL)	0.14 (0.08–0.5)	0.2 (0.1–0.8)	0.3 (0.1–0.7)	0.01
NT-proBNP—day 1 (pg/mL)	2757 (935–7573)	4666 (2948–14,787)	6029 (1334–13,754)	<0.001
D-dimer—day 1 (µg/L)	3470 (1563–4595)	3948 (747–5702)	805 (470–1894)	0.004
Left ventricular ejection fraction (%)	48 (38–55)	44 (30–58)	58 (37–59)	<0.001
Chronic kidney disease (CKD 3, 4, 5)	12 (35%)	5 (33%)	5 (26%)	<0.001
Glomerular filtration rate—day 1	59 (42–72)	44 (29–54)	41 (32–48)	<0.001
Severe course of COVID-19	13 (38%)	9 (60%)	14 (74%)	0.06
Initial saturation—day 1 (%)	92 (90–94)	90 (89–93)	91 (89–95)	0.17
Need for high-flow oxygen	2 (7%)	0	1 (5%)	0.37
Need for intubation	9 (26%)	3 (20%)	3 (16%)	0.69
Pneumonia in computer tomography >50% lung volume	4 (14%)	3 (20%)	1 (5%)	0.44
90 days—mortality	15 (45%)	10 (67%)	13 (68%)	0.004

AF—atrial fibrillation, NT-proBNP—N-terminal prohormone of brain natriuretic peptide.

**Table 3:** Characteristics of patients according to the presence or absence of atrial fibrillation during the index hospitalization.

Characteristics	AF Present during Hospitalization *N* = 51	AF Absent during Hospitalization *N* = 148	*p* Value
Age (years)	75 (70–82)	68 (56–75)	<0.001
Gender (female)	21 (41%)	51 (34%)	0.39
Body mass index (kg/m^2^)	27.62 (26–32.8)	27.9 (24.5–32.8)	0.31
C-reactive protein—day 1 (mg/L)	69.43 (18–129)	52.4 (10–112)	0.58
Interleukin-6—day 1 (pg/mL)	44.79 (21–113)	42.3 (10.6–78)	0.52
White blood cells—day 1 (n/µL)	8.2 (6.4–10.6)	7.2 (5.4–10.5)	0.59
Procalcitonin—day 1 (ng/mL)	0.2 (0.1–0.7)	0.1 (0.06–0.2)	0.61
NT-proBNP—day 1 (pg/mL)	4002 (1334–11,334)	926 (189–2788)	0.13
D-dimer—day 1 (µg/L)	2459 (752–5149)	1318 (691–3470)	0.92
Left ventricular ejection fraction (%)	50 (36–58)	55 (45–60)	0.031
Chronic kidney disease (CKD 3, 4, 5)	16 (31%)	68 (46%)	0.66
Glomerular filtration rate—day 1	46 (35–59)	72 (53–95)	<0.001
Severe course of COVID-19	30 (59%)	68 (46%)	0.11
Initial saturation—day 1 (%)	74 (74–88)	91 (74–97)	<0.001
Need for high-flow oxygen	3 (6%)	12 (8%)	0.59
Need for intubation	12 (24%)	26 (18%)	0.36
Pneumonia in computer tomography >50% lung volume	7 (14%)	11 (7%)	0.19
90 days—mortality	31 (61%)	51 (34%)	0.001

AF—atrial fibrillation, NT-proBNP—N-terminal prohormone of brain natriuretic peptide.

**Table 4:** Univariate predictors of 3-month mortality.

Characteristics	Non-Survivors (*N* = 72)	Survivors (*N* = 127)	*p*-Value
Age (years)	75 (68–82)	67 (55–74)	<0.001
C-reactive protein—day 1 (mg/L)	90 (38–142)	36 (7–99)	<0.001
Interleukin-6—day 1 (pg/mL)	62 (40–168)	29 (8–60)	<0.001
White blood cells—day 1 (n/µL)	9.1 (6.4–12)	6.8 (5.4–9.2)	<0.001
Procalcitonin—day 1 (ng/mL)	0.2 (0.1–0.7)	0.08 (0.06–0.2)	<0.001
NT-proBNP—day 1 (pg/mL)	3181 (1033–10,883)	855 (169–2432)	<0.001
D-dimer—day 1 (µg/L)	2073 (1014–4886)	1236 (628–3721)	0.02
Left ventricular ejection fraction (%)	49.5 (38–58)	57 (47–60)	<0.001
Glomerular filtration rate	43 (33–62)	75 (58–96)	<0.001
History of AF	35 (48%)	33 (26%)	0.001
AF present during hospitalization	29 (40%)	22 (17%)	0.001
AF persistent or permanent	27 (29%)	12 (10%)	<0.001

AF—atrial fibrillation, NT-proBNP—N-terminal prohormone of brain natriuretic peptide.

**Table 5:** Prognostic factors for overall mortality after COVID-19 in multivariate regression model based on history of atrial fibrillation.

Characteristics	*p* Value
Age (years)	<0.001
C-reactive protein—day 1 (mg/L)	0.37
Interleukin-6—day 1 (pg/mL)	0.57
White blood cells—day 1 (n/µL)	0.005
Procalcitonin—day 1 (ng/mL)	0.96
NT-proBNP—day 1 (pg/mL)	0.012
D-dimer—day 1 (µg/L)	0.85
Left ventricular ejection fraction (%)	0.89
Glomerular filtration rate	0.028
History of atrial fibrillation	0.19

NT-proBNP—N-terminal prohormone of brain natriuretic peptide.

**Table 6:** Prognostic factors for overall mortality after COVID-19 in multivariate regression model using categories of permanent and persistent atrial fibrillation vs. absent or paroxysmal form.

Characteristics	*p* Value
Age (years)	0.007
C-reactive protein—day 1 (mg/L)	0.14
Interleukin-6—day 1 (pg/mL)	0.19
White blood cells—day 1 (n/µL)	<0.001
Procalcitonin—day 1 (ng/mL)	0.46
NT-proBNP—day 1 (pg/mL)	0.08
D-dimer—day 1 (µg/L)	0.76
Glomerular filtration rate	0.01
Left ventricular ejection fraction (%)	0.99
Permanent or persistent atrial fibrillation vs. paroxysmal or absent	0.035

NT-proBNP—N-terminal prohormone of brain natriuretic peptide.

**Table 7:** Prognostic factors for overall mortality after COVID-19 in multivariate regression model using the detection of atrial fibrillation.

Characteristics	*p* Value
Age (years)	<0.001
C-reactive protein—day 1 (mg/L)	0.37
Interleukin-6—day 1 (pg/mL)	0.57
White blood cells—day 1 (n/µL)	0.004
Procalcitonin—day 1 (ng/mL)	0.95
NT-proBNP—day 1 (pg/mL)	0.014
D-dimer—day 1 (µg/L)	0.87
Glomerular filtration rate	0.04
Left ventricular ejection fraction (%)	0.73
Atrial fibrillation present during hospitalization	0.15

NT-proBNP—N-terminal prohormone of brain natriuretic peptide.

## Data Availability

The data presented in this study are available on request from the corresponding author due to institutional policy.
